# Effective treatment of liver metastases with photodynamic therapy, using the second-generation photosensitizer meta-tetra(hydroxyphenyl)chlorin (mTHPC), in a rat model

**DOI:** 10.1038/sj.bjc.6690736

**Published:** 1999-10

**Authors:** J P Rovers, A E Saarnak, A Molina, J J Schuitmaker, H J C M Sterenborg, O T Terpstra

**Affiliations:** Departments of Surgery and Ophthalmology, Leiden University Medical Centre, PO Box 9600, Leiden, 2300 RC, The Netherlands; Laser Centre, Academic Medical Centre, PO Box 22700, Amsterdam, 1100 DE, The Netherlands

**Keywords:** photodynamic therapy, photosensitizer, colon cancer, liver metastasis, fluorescence, extraction, tumour response

## Abstract

The only curative treatment for patients with liver metastases to date is surgery, but few patients are suitable candidates for hepatic resection. The majority of patients will have to rely on other treatment modalities for palliation. Photodynamic therapy (PDT) could be a selective, minimally invasive treatment for patients with liver metastases. We studied PDT in an implanted colon carcinoma in the liver of Wag/Rij rats, using the photosensitizer meta-tetra(hydroxyphenyl)chlorin (mTHPC). mTHPC tissue kinetics were studied using ex vivo extractions and in vivo fluorescence measurements. Both methods showed that mTHPC kinetics were different for liver and tumour tissue. After initial high levels at 4 h after administration (0.1 and 0.3 mg kg^−1^) mTHPC in liver tissue decreased rapidly in time. In tumour tissue no decrease in photosensitizer levels occurred, with mTHPC remaining high up to 48 h after administration. Both concentration data and fluorescence data showed an increase in tumour to liver ratios of up to 6.3 and 5.0 respectively. Illumination with 652 nm (15 J) resulted in extensive damage to tumour tissue, with necrosis of up to 13 mm in diameter. Damage to normal liver tissue was mild and transient as serum aspartate aminotransferase and alanine aminotransferase levels normalized within a week after PDT treatment. Long-term effects of mTHPC-PDT were studied on day 28 after treatment. Regardless of drug dose and drug–light interval, PDT with mTHPC resulted in complete tumour remission in 27 out of 31 treated animals (87%), with only four animals in which tumour regrowth was observed. Non-responding tumours proved to be significantly larger (*P* < 0.001) in size before PDT treatment. This study demonstrates that mTHPC is retained in an intrahepatic tumour and that mTHPC-PDT is capable of inducing complete tumour remission of liver tumours. © 1999 Cancer Research Campaign


*British Journal of Cancer* (1999) **81**(4), 600–608
© 1999 Cancer Research Campaign
Article no. bjoc.1999.0736

### Effective treatment of liver metastases with photodynamic therapy, using the second-generation photosensitizer meta-tetra(hydroxyphenyl)chlorin (mTHPC), in a rat model

**JP Rovers[1], AE Saarnak[3], A Molina[1], JJ Schuitmaker[2], HJCM Sterenborg[3] and OT Terpstra[1]**

Departments of [1]Surgery and [2]Ophthalmology, Leiden University Medical Centre, PO Box 9600, 2300 RC Leiden, The Netherlands; [3]Laser Centre, Academic Medical Centre, PO Box 22700, 1100 DE Amsterdam, The Netherlands

**Summary** The only curative treatment for patients with liver metastases to date is surgery, but few patients are suitable candidates for hepatic resection. The majority of patients will have to rely on other treatment modalities for palliation. Photodynamic therapy (PDT) could be a selective, minimally invasive treatment for patients with liver metastases. We studied PDT in an implanted colon carcinoma in the liver of Wag/Rij rats, using the photosensitizer meta-tetra(hydroxyphenyl)chlorin (mTHPC). mTHPC tissue kinetics were studied using ex vivo extractions and in vivo fluorescence measurements. Both methods showed that mTHPC kinetics were different for liver and tumour tissue. After initial high levels at 4 h after administration (0.1 and 0.3 mg $kg^{-1}$) mTHPC in liver tissue decreased rapidly in time. In tumour tissue no decrease in photosensitizer levels occurred, with mTHPC remaining high up to 48 h after administration. Both concentration data and fluorescence data showed an increase in tumour to liver ratios of up to 6.3 and 5.0 respectively. Illumination with 652 nm (15 J) resulted in extensive damage to tumour tissue, with necrosis of up to 13 mm in diameter. Damage to normal liver tissue was mild and transient as serum aspartate aminotransferase and alanine aminotransferase levels normalized within a week after PDT treatment. Long-term effects of mTHPC-PDT were studied on day 28 after treatment. Regardless of drug dose and drug–light interval, PDT with mTHPC resulted in complete tumour remission in 27 out of 31 treated animals (87%), with only four animals in which tumour regrowth was observed. Non-responding tumours proved to be significantly larger ($P < 0.001$) in size before PDT treatment. This study demonstrates that mTHPC is retained in an intrahepatic tumour and that mTHPC-PDT is capable of inducing complete tumour remission of liver tumours. © 1999 Cancer Research Campaign

**Keywords:** photodynamic therapy; photosensitizer; colon cancer; liver metastasis; fluorescence; extraction; tumour response

Colorectal cancer is the third leading cause of cancer death in Western communities. At the time of death approximately two-thirds of patients with colorectal carcinoma will have liver metastases (Welch, 1979). Median survival of untreated patients with liver metastases ranges from 6 to 10 months, mostly depending on the number and size of the metastases (Cady, 1983). Resection of colorectal liver metastases, the only curative treatment to date, is only applicable in 10% of all patients (Ballantyne, 1993). The majority of patients will have to rely on other, mainly palliative treatment modalities, of which none of them have proven to be of real benefit to the patient with irresectable liver metastases (Bush, 1995). Interstitial photodynamic therapy (PDT) could be an effective, minimally invasive treatment for patients with a few liver metastases. PDT is a treatment modality for cancer, in which a photosensitizing drug (photosensitizer) is administered and subsequently illuminated with light of a specific wavelength, matching an absorption peak of the drug. Upon illumination the photosensitizer becomes activated and reacts with available oxygen, causing the production of reactive oxygen species, leading to vascular damage and direct cellular damage (Star et al, 1986; Henderson and Dougherty, 1992). Light used in PDT treatment can be delivered selectively to target tissue via optical fibres placed in the tissue; a treatment called interstitial therapy (Marijnissen et al, 1992). Next to a photochemical reaction, the activated photosensitizer can emit light useful for detection of sensitized tissue (photodiagnostics). In vivo fluorescence measurements can be used to study photosensitizer kinetics non-invasively (Braichotte et al, 1995*a*).

Clinically, PDT is mainly used for treatment of superficially located malignancies, such as lung, skin, bladder, oesophagus, and head and neck cancer (Schuitmaker et al, 1996). It has rarely been used to treat deep-seated malignancies, like liver metastases. The use of PDT for liver neoplasms has been limited as most photosensitizers are efficiently accumulated in normal liver tissue, not leading to selective uptake into malignant tissue. Also, liver tissue, being a highly pigmented tissue, limits deep penetration of light and thus treatment volumes. Experimental studies, using first-generation photosensitizers haematoporphyrin derivative (HpD) and photofrin, have shown PDT to be capable of inducing tumour destruction within the liver (Holt et al, 1985; van Hillegersberg et al, 1992), despite limitations like non-selective uptake and limited light penetration. New, second-generation photosensitizers could possibly establish a more selective accumulation in tumour tissue and, when absorbing at longer wavelength (> 650 nm), could result in larger volumes of necrosis. In a previously performed study we used the photosensitizer bacteriochlorin *a* (BCA), which

*Received 5 August 1998*
*Revised 27 January 1999*
*Accepted 29 January 1999*

*Correspondence to:* OT Terpstra has an absorption maximum at a wavelength of 760 nm (Rovers et al, 1998). Due to deeper penetration of 760 nm light, we were able to induce lesions of up to 16 mm in diameter with a single, plain-cut fibre (diameter 0.4 mm). Although extensive tumour necrosis was induced by BCA-PDT, islands of viable tumour cells remained, leading to tumour regrowth in due time. Because of this we decided to use a potentially more potent photosensitizer, which is meta-tetra(hydroxyphenyl)chlorin (mTHPC).

mTHPC is a single and pure substance with a high absorption peak at a wavelength of 652 nm. Recently, mTHPC has shown to be a very effective photosensitizer in various tumour models and clinical trials (Ris et al, 1991; Lofgren et al, 1994; Peng et al, 1995; Dilkes et al, 1996; Grosjean et al, 1996; Mlkvy et al, 1997), with possible preferential uptake in a colon carcinoma in mice compared to liver concentrations (Whelpton et al, 1995). Furthermore, mTHPC drug and light doses needed to induce tumour necrosis are much lower than that of HpD (Berenbaum et al, 1986).

The aim of this study is to determine mTHPC distribution in tumour and adjacent liver tissue, via tissue extractions and in vivo fluorescence measurements, and to assess short-term and long-term effects of mTHPC-PDT treatment in a rat liver tumour model.

### MATERIALS AND METHODS

### Animals and tumour model

A total of 66 male Wag/Rij rats (Charles River, Sulzfeld, Germany), weighing 200–240 g, were used in these experiments. The animals had free access to food and water. The experiments were approved by the Animal Welfare Committee of the Leiden University Medical Centre and the animals received care in accordance with established guidelines.

We used the CC531 cell line, which is a chemically induced adenocarcinoma of the rat colon, moderately differentiated, syngeneic and transplantable to Wag/Rij rats, for tumour induction in the liver (Marquet et al, 1984). Tumour cells were cultured on RPMI-1640 (Dutch modification) supplemented with 2 mM L-glutamine (Gibco, Grand Island, NY, USA), 10% heat inactivated fetal calf serum, 100 U $ml^{-1}$ penicillin and 0.1 mg $ml^{-1}$ streptomycin sulphate. At laparotomy under inhalation anaesthetics with halothane, $5 \times 10^5$ tumour cells were injected subcapsulary into the liver. For the distribution study, three tumours per rat were induced (left lateral lobe, upper right lobe and lower right lobe), whereas for the PDT efficacy studies one tumour per rat was induced (left lateral lobe). Animals were treated 10 days after tumour cell injection, when tumours had reached a diameter and thickness of 5- to 7-mm.

### Experimental design

In the first part of the study we investigated mTHPC distribution in tumour and liver tissue at different time intervals after intravenous administration. All rats ($n = 20$) were administered 0.3 mg $kg^{-1}$ bodyweight mTHPC via the femoral vein, and they were randomly assigned to four groups. Animals were killed 4, 24, 48 or 72 h after mTHPC administration, after which the liver was removed and tumours were dissected. Tissue samples were immediately frozen in liquid nitrogen and stored at – 20°C until mTHPC analysis was performed.

In the second part of the study we measured in vivo fluorescence levels in tumour and liver tissue after mTHPC administration and, subsequently, determined the effect of interstitial illumination. All animals ($n = 46$) were treated 9 ± 1 days after tumour inoculation. They were randomly assigned to four treatment groups ($n = 10$ per group) and one control group ($n = 6$). Illumination was performed at 4, 24, 48 or 72 h after mTHPC administration. In each treatment group animals received either a dose of 0.1 mg $kg^{-1}$ or 0.3 mg $kg^{-1}$ bodyweight mTHPC, and animals in the control group received either light illumination only or mTHPC administration (0.3 mg $kg^{-1}$) only. At laparotomy, prior to light illumination, in vivo fluorescence measurements were performed on liver and tumour tissue. To measure photosensitizer bleaching, immediately after illumination fluorescence of tumour tissue was determined. Before treatment, tumour sizes were measured using sliding callipers and calculated using the formula: 1/4 π R1 R2, where R1 and R2 are diameters perpendicular to each other.

To qualify short-term effects of PDT treatment, in each treatment group two animals, one of each mTHPC dose, were killed 48 h after illumination ($n = 8$). Sizes of induced damage were measured and livers were sectioned for histological examination. All other animals ($n = 32$) were allowed to survive for 28 days after PDT treatment, to assess long-term effects of PDT treatment. Twenty-eight days after PDT treatment, animals were killed and the livers were removed. Macroscopically, tumour sizes were determined, and microscopically the presence of viable tumour cells was examined to assess tumour response. No viable tumour cells present was considered to be a complete remission (CR), whereas presence of viable tumour cells and tumour growth was considered to be no response (NR) to PDT treatment. To determine serum levels of aspartate aminotransferase (ASAT) and alanine aminotransferase (ALAT) as a parameter of liver damage, blood samples (0.5 ml) were taken by orbital puncture immediately before and 1, 3, 7, 14, 21 and 28 days after PDT treatment.

### Photosensitizer and light delivery mTHPC was kindly donated by Scotia Pharmaceuticals Ltd (Guildford, UK). mTHPC (dry, purple crystals) was dissolved in 20% ethanol (96%), 30% polyethylene glycol (PEG) and 50% water. Animals were kept in subdued light after mTHPC administration to avoid possible side-effects. For light illumination, an argon-pumped dye laser (Spectra Physics Lasers, Mountain View, CA, USA), with sulphorodamine B as dye, was tuned to emit light of 652 nm. Laser light was coupled into two quartz fibres with a core diameter of 0.6 mm, allowing simultaneous illumination of two animals. At laparotomy the liver was mobilized, tumours were exposed and a plain cut fibre was positioned directly onto the tumour surface. Light illumination, with a power output of 100 mW per fibre, was performed for a period of 150 s, delivering an energy of 15 J to each tumour. Tissue fluence rates were not measured in this experiment.

### Fluorescence measurements in vivo

Fluorescence was measured with a setup previously described by Sterenborg et al (1996). In short, a halogen (Hg) lamp was used as light source and the excitation wavelength was 405 nm, selected through an interference filter (Oriel 56541). Excitation light and fluorescence were delivered to and from tissue through a bundle of optical fibres (200 μm) put in contact with the tissue. Fluorescence was detected at two wavelength ranges: red fluorescence (630–750 nm) was detected with a long-pass filter (Schott RG 630) and

© 1999 Cancer Research Campaign 

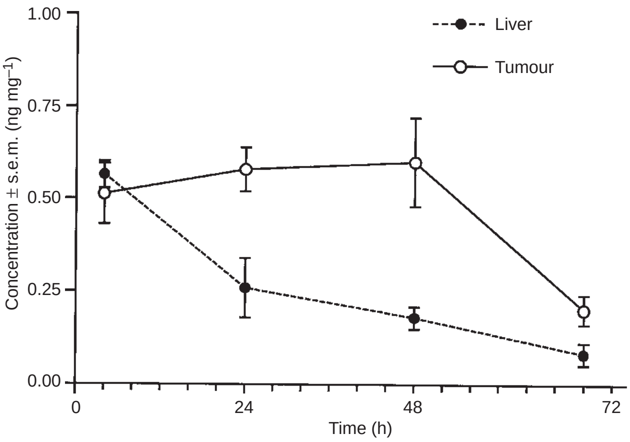

**Figure 1** mTHPC tissue concentrations determined using ex vivo extractions. The graph represents the mean (± s.e.m.) mTHPC concentration in liver and tumour tissue at 4, 24, 48 and 72 h after intravenous administration of mTHPC. Significantly higher mTHPC concentrations were detected in tumour tissue than in liver tissue at 24 ($P = 0.04$) and 48 ($P = 0.006$) hours after drug administration. Difference in tissue concentrations at 72 h after administration was not significant ($P = 0.06$). All values are the mean of five animals, with at least two measurements per tissue per animal a red-sensitive photomultiplier tube (Hamamatsu R 636-10), and autofluorescence (550–600 nm) with a 600 nm cut-off glass filter, a long-pass filter (Schott KV 550) and a green-sensitive photomultiplier tube (Hamamatsu IP 128). A standard lock-in technique was used. A fluorescence ratio (FR) was calculated between the two detected fluorescence intensities to correct measurements for changes in excitation light intensity and measurement geometry. Five measurements were performed per tissue, repositioning the fibre between each measurement, of which the mean ± standard error of the mean (s.e.m.) FRs were calculated.

### mTHPC concentration determination mTHPC concentrations were determined using standard extraction and fluorometry techniques with similarities to the technique as described by Lilge et al (1997). Briefly, frozen tissue samples were weighed and mechanically homogenized in 3-ml dimethyl sulphoxide (DMSO). The homogenate was centrifuged (5000 rpm for 10 min) and fluorescence in the supernatant was determined (excitation 420 nm, emission 650 ± 10 nm) using a standard spectrofluorometer (Aminco SPF 500) and converted into concentration by interpolation in a standard curve constructed with known mTHPC concentrations. After correction for sample weight, mTHPC concentrations were expressed as µg mg$^{-1}$ wet tissue. For each animal the T/L concentration ratio was calculated and per treatment group the mean (± s.e.m.) T/L-ratio was calculated. Note that the mean T/L-ratio can be different from the ratio of the mean tumour and liver tissue concentrations.

### Histological examination

Livers were fixated in a 3.6% buffered formalin solution, sliced through the largest diameter of the tumours, embedded in paraffin wax and sectioned (4 µm). Sections were stained with haematoxylin & eosin (H&E) and examined by a pathologist to detect presence of viable looking tumour cells and to examine induced damage to tumour and surrounding liver tissue.

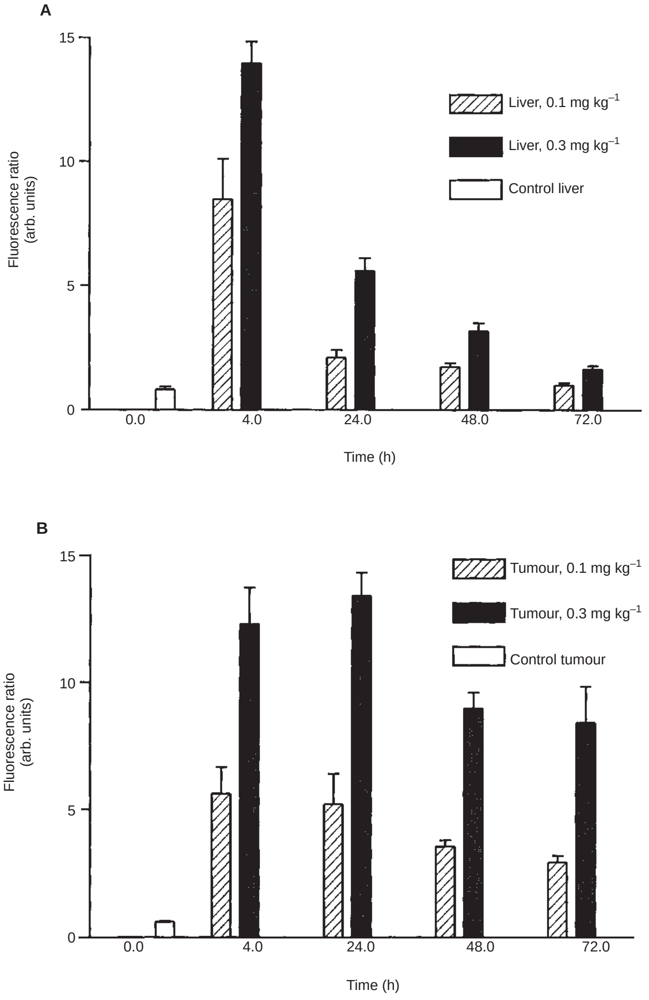

**Figure 2** FR in liver and tumour tissue. FR is the ratio of red fluorescence (630–750 nm) over the autofluorescence (550–600 nm), to compensate for tissue optical properties. The graphs represent the mean (± s.e.m.) FR in (**A**) liver and (**B**) tumour tissue 4, 24, 48, and 72 h after administration of 0.1 or 0.3 mg kg$^{-1}$ mTHPC. In liver tissue there is a significant decline in the FR in time (4–24 h; $P = 0.007$ and $P = 0.001$, 24–48 h; $P = 0.003$ and $P = 0.006$, 48–72 h; $P = 0.006$ and $P = 0.007$, for 0.1 and 0.3 mg kg$^{-1}$ mTHPC respectively). In tumour tissue there was no significant decline in the FR, except for the FR 48 h after 0.3 mg kg$^{-1}$ of mTHPC ($P = 0.005$). FR values are the mean of five animals per treatment group

### Statistical analysis

All values were expressed as mean ± sem. The unpaired Student's *t*-test was used to evaluate differences in mTHPC concentration, fluorescence levels and T/L-ratios between the different time intervals after mTHPC administration and between the two doses of mTHPC used in the experiments. A *P*-value of $< 0.05$ was considered to be statistically significant.

### RESULTS

### mTHPC concentration and in vivo fluorescence measurements

Figure 1 shows that mTHPC concentrations in liver tissue were highest 4 h after administration, with no significant difference

© 1999 Cancer Research Campaign

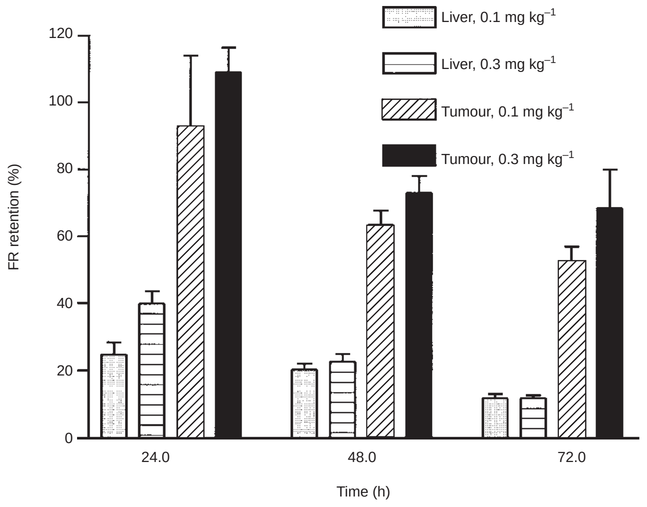

**Figure 3** Retention of fluorescence in liver and tumour tissue. This graph represents FR at 24, 48 and 72 h after mTHPC administration as percentage of the initial FR at 4 h after administration of 0.1 or 0.3 mg $kg^{-1}$. Values represent the mean (± sem) of 5 animals per treatment group. In liver tissue the FR drops rapidly to 25–40% and eventually 12% at 72 h, whereas tumour FRs remain high with 52–68% remaining at 72 h after administration between liver and tumour tissue concentrations, as illustrated by a T/L-ratio of 0.9 ± 0.2 (Table 1). Concentrations in tumour tissue were highest 24 and 48 h after mTHPC administration. mTHPC concentrations in liver tissue decreased rapidly in time, whereas mTHPC concentrations in tumour tissue declined slowly, resulting in a significant difference between liver and tumour mTHPC concentrations at 24 ($P = 0.04$) and 48 ($P = 0.006$) h after administration. The mean T/L-ratio increased up to 6.3 at 72 h after mTHPC administration.

In vivo fluorescence measurements showed comparable results to extraction data, as illustrated in Figure 2; highest FR in liver tissue were found 4 h after mTHPC administration with a rapid decrease in time. From 24 h on, FRs were significantly higher ($P < 0.01$) in tumour tissue than in liver tissue. The FRs in tumour tissue remained high, whereas in liver tissue they decreased rapidly, as clearly indicated by retention of fluorescence (Figure 3). Retention represents the FR as a percentage of FR measured at 4 h after mTHPC administration. Retention in tumour tissue was 93% and 109%, for 0.1 and 0.3 mg $kg^{-1}$ mTHPC respectively, 24 h after administration and decreases to 52% and 68% at 72 h, whereas retention in liver tissue was 25% and 40% at 24 h and 12% for both drug doses at 72 h after administration. The T/L-ratios of fluorescence data were comparable to concentration ratios, as shown in Table 1.

In all treatment groups a decrease in tumour FR was seen immediately after illumination (bleaching), with an overall bleaching percentage between 60% and 75% and there was no significant difference in percentage of bleaching between the different treatment groups, except for animals illuminated 72 h after injection of 0.1 mg $kg^{-1}$ mTHPC, where bleaching was only 41% (Figure 4).

### Short-term PDT effect

On histological examination sharply demarcated lesions were seen with extensive necrosis of tumour tissue and some necrosis of surrounding liver tissue. The largest diameters of PDT-induced necrosis were measured in the animals treated 4 h after administration of 0.1 or 0.3 mg $kg^{-1}$, 10 and 13 mm respectively. PDT at later time intervals after drug administration resulted in smaller lesions, ranging from 7 to 9 mm. PDT-induced lesions at later time intervals were more comparable to tumour sizes before treatment and it seems that damage at these time intervals was more restricted to the tumour area (Table 2).

Histological examination of tumours treated with PDT showed in some at random sections the presence of islands of viable looking tumour cells (Figure 5A). Invasion of granulocytes and macrophages was seen in all sections, indicating the occurrence of an acute inflammatory response.

Directly after PDT treatment both serum ASAT and ALAT levels rose, as represented in Figure 6. Rise in serum enzyme levels was more profound upon illumination 4 h after mTHPC administration than upon illumination 48 or 72 h after administration, indicating that the extent of PDT induced liver damage was highest at earlier time points between drug administration and illumination. Serum ASAT and ALAT levels normalized within a week after PDT treatment.

### Long-term PDT effect

Assessment of tumour response, 28 days after PDT treatment, showed complete remissions in 27 out of 31 treated animals (87%), with only four animals in which PDT treatment had no effect (Table 3). Tumour sizes of non-responding animals were comparable to tumour sizes of control animals. Although there was no significant difference in tumour size before PDT treatment between different treatment groups, non-responding tumours

**Table 1** Tumour to liver ratio after intravenous injection of mTHPC

| T/L-ratio Drug–light interval (h) | Fluorescence measurements | | Concentration measurements | |
|---|---|---|---|---|
| | 0.1 mg $kg^{-1}$ | 0.3 mg $kg^{-1}$ | 0.1 mg $kg^{-1}$ | 0.3 mg $kg^{-1}$ |
| 4 | 0.7 ± 0.1 | 0.9 ± 0.1 | – | 0.9 ± 0.2 |
| 24 | 2.5 ± 0.5 | 2.5 ± 0.3 | – | 2.6 ± 0.5 |
| 48 | 2.1 ± 0.1 | 2.9 ± 0.2 | – | 3.7 ± 0.7 |
| 72 | 3.1 ± 0.5 | 5.0 ± 0.5 | – | 6.3 ± 2.7 |

The mean (± s.e.m.) T/L-ratios were calculated using ex vivo extraction data and in vivo fluorescence data (FR). For in vivo fluorescence measurements 0.1 and 0.3 mg $kg^{-1}$ mTHPC was administered, whereas for concentration determinations only 0.3 mg $kg^{-1}$ mTHPC was given. All values are the mean of five animals.

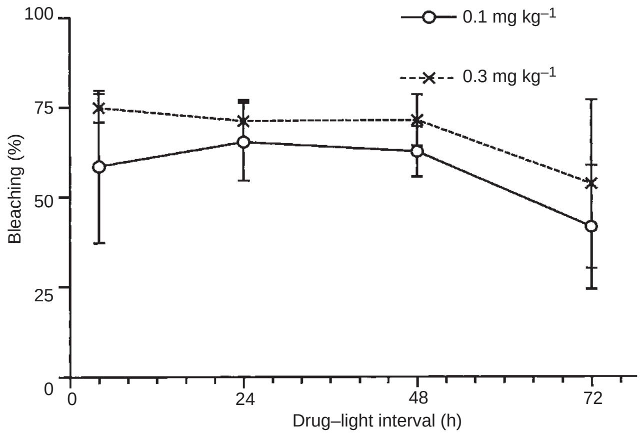

**Figure 4** Photosensitizer bleaching in tumour tissue. This graph shows the mean (± s.e.m.) percentage of FR decrease in tumour tissue after illumination with 652 nm, representing photobleaching of mTHPC. There is no significant difference in bleaching between treatment groups, with values ranging from 60 to 72%, except for 72 h after administration of 0.1 mg $kg^{-1}$, where bleaching is only 41%. Values are the mean of five animals per treatment group proved to be significantly larger in size before PDT treatment than responding tumours, with mean tumour sizes of 37.0 ± 8.5 $mm^2$ and 24.7 ± 9.6 $mm^2$ respectively ($P < 0.001$).

On histological examination, in case of a CR, only a small fibrotic lesion was visible on the site where the tumour had been (Figure 5B). There was a sharp demarcation between healthy and PDT-damaged tissue, with the occurrence of liver regeneration at the border; a proliferation of bile ducts was seen as well as proliferation of hepatocytes. In fibrotic lesions different zones could be identified: in the centre necrotic tissue, surrounded by a rim of granulocytes and lymphocytes, which was surrounded by a rim of macrophages (Figure 5C). Non-responding tumours did not show a difference in morphology compared to non-treated tumours.

### DISCUSSION

PDT has the potential of selectively destroying malignant tissue with minimal damage to healthy tissue. Selectivity of PDT depends on both photosensitizer localization in tissue and light administration, which makes it important to determine photosensitizer distribution in target tissue and its surrounding tissue. In case

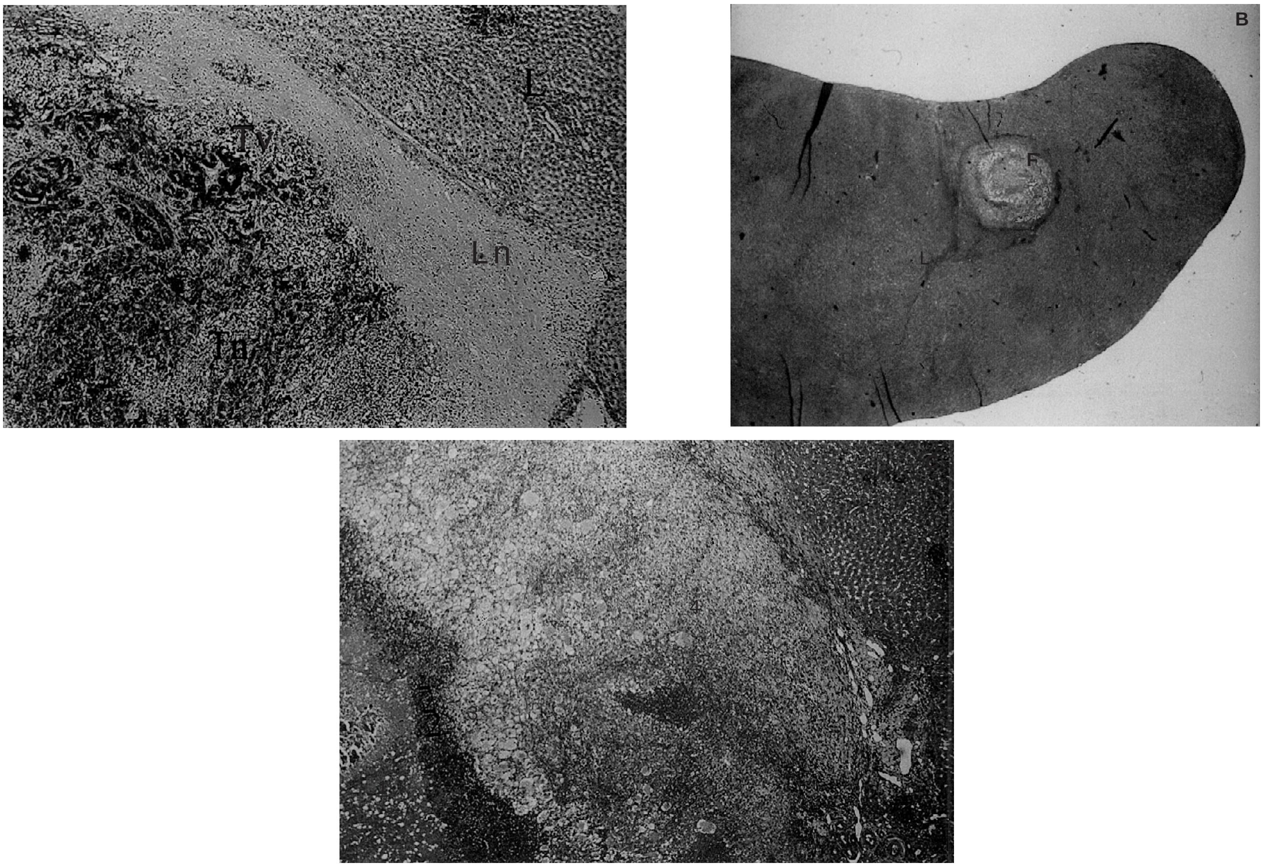

**Figure 5** (**A**) Histological section of a PDT-treated tumour, 48 h after illumination. The animal was illuminated with 15 J of 652 nm light 4 h after administration of 0.3 mg $kg^{-1}$ of mTHPC. Extensive tumour necrosis (Tn) and necrosis of a rim of normal liver tissue (Ln) is seen in the liver (L). Islands of viable looking tumour cells (Tv) can be identified within the treated area. **B, C** Histological section of PDT treated tumours on day 28 after illumination. Animals were treated with 15 J of laser light 24 (**A**) or 48 (**B**) h after administration of 0.3 mg $kg^{-1}$ of mTHPC. (**B**) Only a small fibrotic lesion (FI) remained on the site where a tumour had been, surrounded by normal liver tissue (L). (**C**) In fibrotic lesions (4) in the liver (5) different zones could be identified: (1) central necrosis, surrounded by (2) a rim of granulocytes and lymphocytes, which was surrounded by (3) a rim of macrophages

 *© 1999 Cancer Research Campaign*

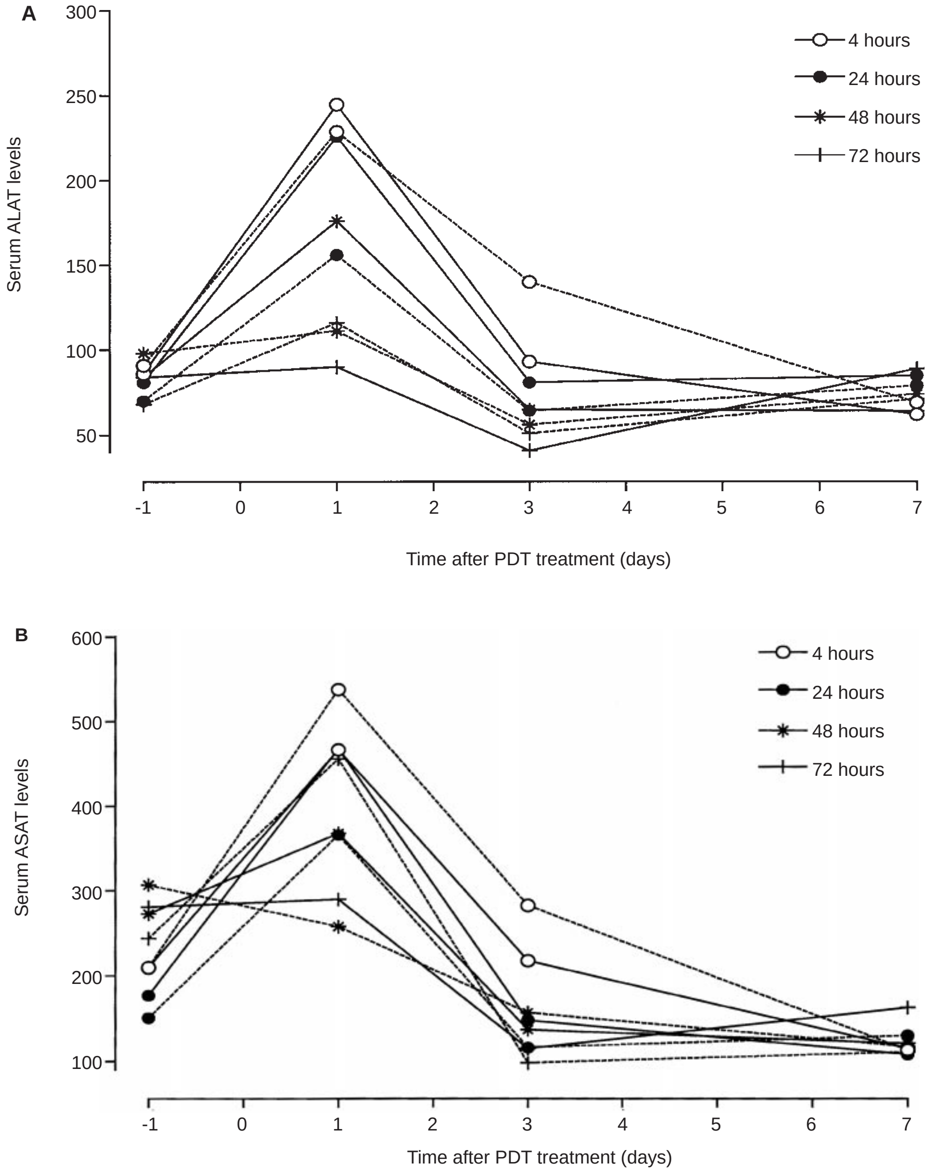

**Figure 6** Changes in serum enzyme levels after PDT treatment of liver metastases in rats. The graph shows changes in serum (**A**) ALAT and (**B**) ASAT levels up to seven days after PDT treatment with (- - -) 0.1 or (—) 0.3 mg kg$^{-1}$ mTHPC. Each point represents the mean of five animals

**Table 2** Tumour response after mTHPC-PDT 48 h after light delivery

| | Area of PDT damage (mm$^2$)[a] | | | |
|---|---|---|---|---|
| **Time (h)**[b] | **0.1 mg kg$^{-1}$**[c] | | **0.3 mg kg$^{-1}$**[c] | |
| 4 h | 79.3 | *39.4* | 93.7 | *21.6* |
| 24 h | 28.3 | *30.1* | 49.1 | *26.9* |
| 48 h | 27.6 | *15.9* | 32.0 | *14.1* |
| 72 h | 41.0 | *31.0* | 27.5 | *23.9* |

The area (mm$^2$) of PDT damage, measured 48 hours after PDT treatment, are given for each drug dose ($n$ = 1) and time interval (n = 1) after mTHPC administration. [a]Figures in italics represent tumour sizes (mm$^2$) before PDT treatment. [b]Time between drug administration and light delivery (h). [c]Administered dose of mTHPC via the femoral vein.

of a tumour in a highly vascularized organ as the liver, it will be difficult to reach selective drug uptake, as most photosensitizers are efficiently accumulated in liver tissue (Bown et al, 1986; Bellnier et al, 1989). Only for endogenously generated protoporphyrin-IX, after aminolaevulinic acid (ALA) administration, tumour selectivity has been reported, with tumour to liver ratio of 4:1 (Hillegersberg et al, 1992).

We studied the mTHPC distribution in a transplanted colon adenocarcinoma in a rat liver at different times after intravenous administration, using ex vivo tissue extractions and in vivo fluorescence measurements. Both methods showed different mTHPC pharmacokinetics in liver and tumour tissue. In time, mTHPC concentrations in liver tissue decreased rapidly, whereas mTHPC in tumour tissue remained high up to 48 h after injection. As previously reported by

**Table 3** Tumour response after mTHPC-PDT 28 days after light delivery

| | Complete remission[b] | | No remission[b] | |
|---|---|---|---|---|
| **Drug-light interval (h)** | **0.1 mg kg$^{-1}$** | **0.3 mg kg$^{-1}$** | **0.1 mg kg$^{-1}$** | **0.3 mg kg$^{-1}$** |
| 4 | 3/4 | 3/4 | 1/4 | 1/4 |
| 24 | 4/4 | 3/3 | – | – |
| 48 | 4/4 | 3/4 | – | 1/4 |
| 72 | 4/4 | 3/4 | – | 1/4 |
| Control[a] | – | | 3/3 | |

Illumination (= 652 nm, 15 J) was performed 4, 24, 48 and 72 h after intravenous administration of either 0.1 or 0.3 mg kg$^{-1}$ mTHPC. No viable tumour cells upon histological examination was considered to be a complete remission (CR), whereas tumour growth was considered to be no remission (NR). [a]Control group received mTHPC administration only. [b]No. of tumours in complete remission/no. of animals.

Whelpton (Whelpton et al, 1995, 1996), in liver tissue mTHPC showed an initial rapid decline in the first hours after administration, followed by a slow decline. We observed similar kinetics in liver tissue for BCA (Rovers et al, 1998).

In vivo fluorescence measurements have been used as a minimally invasive method to study photosensitizer pharmacokinetics in animals and humans (Alian et al, 1994; Braichotte et al, 1995*b*). However, a problem associated with fluorescence measurements is the difficulty of obtaining quantitative fluorophore concentrations, due to varying optical properties of tissues. This makes comparison of fluorescence intensities between tissue types difficult, especially between dark red liver tissue and pale tumour tissue; as absorption in liver tissue is higher than in tumour tissue, less light is transmitted back for fluorescence measurements, possibly leading to underestimation of fluorophores in liver tissue compared to those in tumour tissue. Use of the FR corrects partially for differences in optical properties as fluorescence values are divided by the autofluorescence, making comparison of the FR between two tissue types more reliable. Comparison of fluorescence levels within the same organ is not hindered by difference in optical properties and thus seems a reliable method to study in vivo photosensitizer kinetics.

In vivo fluorescence measurements showed similar mTHPC tissue kinetics as concentration data; FR in liver tissue rapidly declined in time, to only 12% of the initial value measured at 4 h after administration, confirming findings of Alian et al (1994). While liver tissue FR decreased in time, tumour tissue showed no significant decrease in FRs, leading to significantly higher FR levels in tumour tissue with a mean T/L-ratio of up to 5.0 ± 0.5 at 72 h after mTHPC administration. A similar increase in T/L-ratios, up to 6.3 ± 2.7 at 72 h after administration, was seen using concentration data. In vivo fluorescence measurement showed to be a useful, non-invasive technique to study drug pharmacokinetics and the use of the FR allowed tissue comparisons. Both ex vivo extractions and in vivo fluorescence measurements showed a selective retention of mTHPC in tumour tissue, highest 3 days after drug administration.

FRs in tumour tissue dropped to 25–40% of the initial value after illumination, which is caused by photosensitizer bleaching. Providing mTHPC does not produce toxic products on bleaching, strong bleaching of mTHPC could be advantageous at drug threshold levels, at which sensitizer levels in normal tissue are low enough to be totally bleached before inducing toxicity. Some even propose that precise dosimetry is not essential, when using a highly bleachable photosensitizer (Potter et al, 1987). Photobleaching could be used to provide a real-time indication of the PDT effect upon treatment (Wilson et al, 1997).

PDT with mTHPC was capable of inducing complete tumour destruction of transplanted tumours within the liver. Although a zone of liver tissue is damaged around the illuminated tumour, liver damage is minimal and transient as serum enzyme levels of ASAT and ALAT normalize within a week after treatment. Normal tissue damage is limited by: (1) local light administration using optical fibres; (2) strong absorption of light in liver tissue, limiting light penetration; and (3) strong bleaching of mTHPC at threshold levels, which will be the case at longer drug-light intervals. mTHPC-PDT of liver tumours resulted in an overall CR rate of 87%, with only four out of the 31 treated animals in which tumour regrowth occurred. Tumour regrowth seemed to be the result of insufficient tumour illumination, as tumour sizes before PDT treatment were significantly larger in these animals. Using a single, plain-cut fibre we were able to reach a 100% CR of all tumours less than 30 mm$^2$ in size. Optimizing tumour illumination, by using cylindrical diffusers and multiple fibres, will insure a more homogenous light administration over larger areas, enabling effective treatment of larger tumour volumes (Mizeret et al, 1996).

A drug dose of 0.1 mg kg$^{-1}$ mTHPC and a light dose of 15 J was sufficient to effectively treat liver tumours in the rat model, stating mTHPC's potency. Although PDT has proven to be effective in tumour destruction within the liver using haematoporphyrin derivative (Holt et al, 1985), photofrin (van Hillegersberg et al, 1992), pheophorbide a (Nishiwaki et al, 1989) and ALA (Svanberg et al, 1996), much higher light and drug doses were needed. This is illustrated for photofrin in a liver tumour model in rats: best results were obtained at a light dose of 800 J cm$^{-1}$, with complete remission of four out of six tumours (van Hillegersberg et al, 1992). Our study clearly indicates that mTHPC is much more potent than Photofrin, resulting in complete remissions at lower light doses and, consequently, short treatment times. This makes mTHPC one of the most potent photosensitizer currently available for treatment of intrahepatic tumours.

Based on our results, we were not able to determine an optimal drug–light interval for mTHPC, as treatment at each time point resulted in complete remissions. Illumination shortly after mTHPC administration is feasible, as liver damage is minimal and drug levels in tissue are high. PDT in this case will mainly rely on vascular damage and less on direct cellular damage. However, as drug levels in tumour surrounding liver tissue are high, light delivery, and thus fibre placement, needs to be accurate. A practical advantage would be that drug injection and light illumination

 © 1999 Cancer Research Campaign could be performed the same day, limiting hospitalization times. On the other hand, treatment at later time intervals will limit damage to surrounding liver tissue even further, based on drug-induced tumour selectivity, making accurate fibre placement less important. We believe drug selectivity is less important, as damage to a rim of normal liver tissue is tolerated and even preferred in treatment of cancerous tissue. Of utmost importance is presence of enough photosensitizer in tumour tissue to effectively eradicate tumour cells. Illumination should thus be performed when tumour tissue concentrations are highest, which is at later time points after mTHPC administration.

Like many other tissues, liver tissue heals mainly by regeneration after PDT treatment, which is apparent by bile duct proliferation and hepatocyte proliferation. In case of CR, only a fibrotic lesion remained at the site where a tumour had been, with liver regeneration at its border. Different zones could be identified within the lesion with (1) a central necrotic part, surrounded by (2) a zone of granulocytes and (3) a zone of macrophages. The presence of these cells confirm the occurrence of a non-specific immune response upon PDT treatment, with activation and accumulation of host immune cells (Korbelik and Krosl, 1994; de Vree et al, 1996). We observed the presence of some viable looking tumour cells at histological examination 2 days after PDT treatment, though in the same treatment group all animals had CR, 28 days after PDT treatment. An explanation for this could well be effective destruction of remaining tumour cells by the PDT-elicited immune response.

In conclusion, mTHPC was retained in tumour tissue, leading to tumour selectivity in time. Illumination of sensitized tumours resulted in CR of all tumours less than 30 $mm^2$ in size, without inducing severe liver damage. Drug doses and light doses used for mTHPC-PDT were far less than needed with other photosensitizers, making mTHPC the most potent photosensitizer currently available for treatment of intrahepatic tumours. In patients, light delivery can be performed percutaneously using laser fibres positioned in the tumour under ultrasound or computerized tomography (CT), as is being done in laser photocoagulation (Amin et al, 1993). Feasibility of interstitial PDT (IPDT) has been demonstrated by Purkiss et al (1993). The aim of IPDT for liver metastases will at first be palliative treatment of patients with few irresectable metastases. A clinical study is in progress to assess safety and effect of IPDT with mTHPC in treatment of colorectal liver metastases.

### ACKNOWLEDGEMENTS

We like to thank Dr H van Krieken for his help on the histological data and Rob Keijzer for his help with preparation of histological materials. We like to thank Mrs HAM Holtslag, M van Brakel and T van der Nat for their technical assistance. mTHPC was kindly donated by Scotia Pharmaceuticals, Guildford, UK.

### REFERENCES

Alian W, Andersson-Engels S, Svanberg K and Svanberg S (1994) Laser-induced fluorescence studies of meso-tetra(hydroxyphenyl)chlorin in malignant and normal tissue in rats. *Br J Cancer* **70**: 880–885

Amin Z, Donald JJ, Masters A, Kantr R, Steger AC, Bown SG and Lees WR (1993) Hepatic metastases interstitial laser photocoagulation with real-time US-monitoring and dynamic CT evaluation of treatment. *Radiology* **187**: 339–347

Ballantyne GH and Quin J (1993) Surgical treatment of liver metastases in patients with colorectal cancer. *Cancer* **71**: 4252–4266

Bellnier DA, Ho YK, Pandey RK, Missert JR and Dougherty TJ (1989) Distribution and elimination of Photofrin II in mice. *Photochem Photobiol* **50**: 221–228

Berenbaum MC, Akande SL, Bonnet R, Kaur H, Ioannou S, White RD and Winfield UJ (1986) meso-Tetra-(hydroxyphenyl) porphyrins, a new class of potent tumour photosensitizers with favourable selectivity. *Br J Cancer* **54**: 717–725

Bown SG, Tralau CJ, Coleridge-Smith PD, Akdemir D and Wieman TJ (1986) Photodynamic therapy with porphyrin and phthalocyanine sensitization: quantitative studies in normal rat liver. *Br J Cancer* **54**: 43–52

Braichotte D, Savary JF, Glanzman T, et al. (1995*a*) Clinical pharmacokinetic studies of tetra(meta-hydroxyphenyl)chlorin in squamous cell carcinoma by fluorescence spectroscopy at 2 wavelengths. *Int J Cancer* **63**: 198–204

Braichotte D, Wagnieres GA, Bays R, Monnier P and van den Bergh HE (1995*b*) Clinical pharmacokinetic studies of photofrin by fluorescence spectroscopy in the oral cavity, the esophagus, and the bronchi. *Cancer* **75**: 2768–2778

Bush E and Kemeny MM (1995) Colorectal cancer: hepatic-directed therapy – role of surgery, regional chemotherapy, and novel modalities. *Semin Oncol* **22**: 494–508

Cady B (1983) Natural history of primary and secondary tumors of the liver. *Semin Oncol* **10**: 127–133

de Vree WJA, Esser MC, De Bruijn HS, Star WM, Koster JF and Sluiter W (1996) Evidence for an important role of neutrophils in the efficacy of photodynamic therapy in vivo. *Cancer Res* **56**: 2908–2911

Dilkes MG, Dejode ML, Rowntree-Taylor A, McGilligan JA, Kenyon GS and McKelvie P (1996) m-THPC photodynamic therapy for head and neck cancer. *Lasers Med Sci* **11**: 23–29

Fingar VH, Wieman TJ and Doak KW (1990) Role of thromboxane and prostacyclin release on photodynamic therapy-induced tumor destruction. *Cancer Res* **50**: 2599–2603

Grosjean P, Savary JF, Wagnieres G, Mizeret J, et al. (1996) Tetra(m-hydroxyphenyl)chlorin clinical photodynamic therapy of early bronchial and oesophageal cancers. *Lasers Med Sci* **11**: 227–235

Henderson BW and Dougherty (1992) How does photodynamic therapy work? *Photochem Photobiol* **55**: 145–157

Holt S, Tulip J, Hamilton D, Cummins J, Fields A and Dick C (1985) Experimental laser phototherapy of the morris 7777 hepatoma in the rat. *Hepatology* **5**: 175–180

Korbelik M and Krosl G (1994) Enhanced macrophage cytotoxicity against tumor cells treated with photodynamic therapy. *Photochem Photobiol* **60**: 497–502

Lilge L, O'Carroll C and Wilson BC (1997) A solubilization technique for photosensitizer quantification in ex vivo tissue samples. *J Photochem Photobiol B* **39**: 229–235

Lofgren LA, Ronn AM, Abramson AL, Shikowitz MJ, Nouri M, Lee CJ, Batti J and Steinberg BM (1994) Photodynamic therapy using m-tetra(hydroxyphenyl)-chlorin. An animal model. *Arch Otolaryngol Head Neck Surg* **120**: 1355–1362

Marijnissen JP, Versteeg JA, Star WM and van Putten WL (1992) Tumor and normal tissue response to interstitial photodynamic therapy of the rat R-1 rhabdomyosarcoma. *Int J Radiat Oncol Biol Phys* **22**: 963–972

Marquet RL, Westbroek DL and Jeekel J (1984) Interferon treatment of a transplantable rat colon adenocarcinoma: importance of tumor site. *Int J Cancer* **33**: 689–692.

Mizeret JC and van den Bergh HE (1996) Cylindrical fiberoptic light diffuser for medical applications. *Lasers Surg Med* **19**: 159–167

Mlkvy P, Messman H, MacRobert AJ, Pauer M, Sams VR, Davies CL, Stewart JCM and Bown SG (1997) Photodynamic therapy of a transplanted pancreatic cancer model using meta-tetrahydroxyphenylchlorin (mTHPC). *Br J Cancer* **76**: 713–718

Nishiwaki Y, Nakamura S and Sakaguchi S (1989) New method of photosensitizer accumulation for photodynamic therapy in an experimental liver tumour. *Lasers Surg Med* **9**: 254–263

Peng Q, Moan J, Ma LW and Nesland JM (1995) Uptake, localization, and photodynamic effect of meso-tetra(hydroxyphenyl)porphyrin and its corresponding chlorin in normal and tumour tissue of mice bearing mammary carcinoma. *Cancer Res* **55**: 2620–2626

Potter WR, Mang TS and Dougherty TJ (1987) The theory of photodynamic therapy dosimetry: consequences of photodestruction of sensitizers. *Photochem Photobiol* **46**: 97–101

Purkiss SF, Grahn MF, Abulafi AM, Dean JT, Allardice JT and Williams NS (1994) Multiple fibre interstitial photodynamic therapy of patients with colorectal liver metastases. *Lasers Med Sci* **9**: 27–35

Ris HB, Altermatt HJ, Inderbitzi R, Hess R, et al. (1991) Photodynamic therapy with chlorins for diffuse malignant mesothelioma: initial clinical results. *Br J Cancer* **64**: 1116–1120

© 1999 Cancer Research Campaign 

Rovers JP, Schuitmaker JJ, Vahrmeijer AL, Van Dierendonck JH and Terpstra OT (1998) Interstitial photodynamic therapy with the second generation photosensitizer bacteriochlorin in a rat model for liver metastases. *Br J Cancer* **77**: 2098–2103

Schuitmaker JJ, Baas P, van Leengoed HLLM, van der Meulen FW, Star WM and van Zandwijk N (1996) Photodynamic therapy: a promising new modality for treatment of cancer. *J Photochem Photobiol B: Biology* **34**: 3–12

Star WM, Marijnissen HP, van den Berg-Blok AE, Versteeg JA, Franken FA and Reinhold HS (1986) Destruction of rat mammary tumor and normal tissue microcirculation by hematoporphyrin derivative photoradiation observed in vivo in sandwich observation chambers. *Cancer Res* **46**: 2532–2540

Sterenborg HJ, Saarnak AE, Frank R and Motamedi M (1996) Evaluation of spectral correction techniques for fluorescence measurements on pigmented lesions in vivo. *J Photochem Photobiol B: Biology* **35**: 159–165

Svanberg K, Liu DL, Wang I, Andersson-Engels S, Stenram U and Svanberg S (1996) Photodynamic therapy using intravenous d-aminolaevulinic acid-induced protoporphyrin IX sensitisation in experimental hepatic tumours in rats. *Br J Cancer* **74**: 1526–1533

van Hillegersberg R, Marijnissen JPA, Kort WJ, Zondervan PE, Terpstra OT and Star WM (1992a) Interstitial photodynamic therapy in a rat liver metastases model. *Br J Cancer* **66**: 1005–1014

van Hillegersberg R, van den Berg JW, Kort WJ, Terpstra OT and Wilson JH (1992b) Selective accumulation of endogenously produced porphyrins in a liver metastasis model in rats. *Gastroenterology* **103**: 647–651

Welch JP and Donaldson GA (1979) The clinical correlation of an autopsy study of recurrent colorectal cancer. *Ann Surg* **89**: 496–502

Whelpton R, Michael-Titus AT, Basra SS and Grahn MF (1995) Distribution of temoporfin, a new photosensitizer for the photodynamic therapy of cancer, in a murine tumor model. *Photochem Photobiol* **61**: 397–401

Whelpton R, Michael-Titus AT, Jamdar RP, Abdillah K and Grahn MF (1996) Distribution and excretion of radiolabelled temoporphin in a murine tumour model. *Photochem Photobiol* **63**: 885–891

Wilson BC, Patterson MS and Lilge L (1997) Implicit and explicit dosimetry in photodynamic therapy: a new paradigm. *Lasers Med Sci* **12**: 182–199

*© 1999 Cancer Research Campaign*
